# Sex and Eating: Relationships Based on Wanting and Liking

**DOI:** 10.3389/fpsyg.2015.02044

**Published:** 2016-01-11

**Authors:** Ying Kang, Lijun Zheng, Yong Zheng

**Affiliations:** School of Psychology, Southwest UniversityChongqing, China

**Keywords:** sex, eating, relationship, wanting, liking, model, LPA

## Abstract

Sex and eating may have behavioral and psychological relationships and have cortical regions in common. This research investigated the general relationship between sex and eating from a reward perspective among the general population. Two-hundred and sixty-one Chinese participants were recruited via the internet (136 males, 125 females, mean age 30.46 years) to fill in questionnaires about wanting and liking for sex and eating. The results revealed that first, there was a positive correlation between wanting for sex and wanting to eat only for males. Second, the relationship between liking for sex and eating was also positive for males and not significant in females. Third, the correlation between sociosexual orientation and wanting to eat was significant only in females, and there was no significant correlation between sociosexual orientation and liking for eating. Fourth, emotional sex cravings (or emotional sexual activity) was positively correlated with emotional food cravings (or emotional eating behavior), with a higher magnitude correlation in males than females. Finally, analysis of wanting (liking) models of sex and eating for males and females revealed three models for wanting among females: high wanting, low wanting for eating, and low wanting for sex; and two models for wanting among males: high wanting and low wanting. Liking for sex and eating among females consisted of two types of model: high liking and low liking; whereas three type models existed for males: high liking for sex, high liking for eating, and low liking. In general, our research revealed that, as with other natural reward, sex and eating have considerable commonality and are related in numerous ways.

## Introduction

In Chinese culture, the concepts of food and sex are traditionally linked, and many expressions describe food and sex in terms of each other, such as “a beauty to feast one’s eyes on” and “when the belly is full, the mind is among the maids.” There is also the saying that people who like eating have a high sex drive; or, conversely, people who have little desire to eat have little desire for sex. Sex and eating are natural, instinctive needs: the former encourages reproduction, ensuring continuation of the species, whereas the latter is required for individual survival.

Existing research has illuminated the relationship between eating and sex. For example, Van den Bergh et al. (2008) found that males exposed to sexual pictures showed increased impatience for candy bars and cans of soda pop. That is, exposure to sexual reward stimuli enhances the desire for food reward stimuli. However, this research (Van den Bergh et al., 2008) also found that exposure to sexual pictures increased the impatience for money reward, which indicated exposure to sexual reward stimulus could enhance the desire for another reward stimulus, not only for food reward stimulus. Therefore, we wonder whether there is any relationship specifically between sex and eating. Kniffin and Brian (2012) found that when participants’ current romantic partner was contacted by a previous romantic partner, sharing a meal elicited more sexual jealousy than interactions that do not involve eating, such as talking on the phone, e-mail contact, or having coffee. They demonstrated that for romantic partners, sharing food involves “sharing more than just food.” In addition, eating disorders seem to be associated with abnormal sexual behavior, non-traditional sex roles, and non-heterosexual orientation. First, pathological eating disorders are associated with abnormal sexual behavior. Compared with controls, females with bulimia nervosa exhibit more sexual activity and report greater sexual experience (Culbert and Klump, 2005), while females with anorexia nervosa have decreased sexual interest, lower scores on the Female Sexual Functional Index subscale, and more disturbed sexual function (Tuiten et al., 1993; Gonidakis et al., 2015). Second, masculinity has a negative relationship with eating pathology in men (Blashill, 2011). Compared with heterosexual people, “mostly heterosexuals,” bisexuals, and homosexuals are more likely to report binge eating (Austin et al., 2009). Males who have more sex partners (male and/or female) and more same-gender sex partners are more likely to have specific disordered eating behaviors (Ackard et al., 2008). Finally, the brain imaging literature reports that eating and sex share some brain areas (Georgiadis and Kringelbach, 2012).

Both life experiences and scientific research have indicated a link between sex and eating. However, little research has directly explored this relationship. Although the correlation between sex and eating can be inferred from studies focusing on eating disorders and sexuality, whether there exists any general relationship between sex and eating in a community sample remains unclear. Therefore, in this study we explored this possibility.

Herzog et al. (1984) found gender differences in the relationship between eating disorders and abnormal sexuality, and many studies have found gender differences in sex and eating (Rolls et al., 1991; Oliver and Hyde, 1993; Baumeister et al., 2001; Striegel-Moore et al., 2009). As such, we also explored gender differences in the correlation between sex and eating.

According to incentive salience theory, reward consists of two components, i.e., wanting and liking. Wanting is the motivation to approach stimuli, whereas liking refers to the pleasure derived from stimuli (Berridge, 1996, 2009; Berridge et al., 2009). The two components are dissociable and may be controlled by different brain mechanisms (Robinson and Berridge, 1993; Berridge and Kringelbach, 2008; Berridge, 2009). That the two components are separate has been found in research on food (Berridge, 1996, 2009; Finlayson et al., 2007; Havermans, 2011), alcohol (Hobbs et al., 2005), smoking (Brauer et al., 2001), drug use (Brauer and De Wit, 1997; Breiter et al., 1997), and sex (Krishnamurti and Loewenstein, 2012; Dewitte, 2015). Therefore, we explored the relationship between sex and eating from the perspective of wanting and liking.

We first asked whether there is a correlation between wanting for sex and wanting for eating, between liking for sex and liking for eating, and whether there exist gender differences in these relationships. We also analyzed the relationships between sociosexual orientation and wanting/liking for eating. Second, as mentioned above, much prior research has found a correlation between eating disorders and non-traditional sex roles, and non-heterosexual orientation. Therefore, we investigated the relationship between sex and eating during a negative emotional state. Sex and eating both can be influenced by negative mood or stress (Bancroft et al., 2003a,b; Hamilton and Meston, 2011; Cardi et al., 2015), but the relationship between sex and eating under stress remains relatively unknown. Therefore, we also addressed the relationship between emotional sex cravings (and sexual activity) and emotional food cravings (and eating behavior), to further understand the relationship between sex and eating. Further, we explored gender differences. Finally, we asked whether the statement “people who like eating have high sex drive” can be supported empirically and we considered whether an individual who receives intense pleasure from sex also derives comparable pleasure from eating. We analyzed models of wanting (liking) for sex and eating, whereby individuals were classified into different wanting (liking) groups according to their different levels of wanting (liking) for sex and eating. Given the aforementioned gender differences in eating and sexuality, the model analyses were conducted separately by gender. Further, because sex and eating are two of the most important events of live beings, we were also curious about whether there was any difference in demographic characteristics among different wanting (liking) groups. So we explored age, height, weight, and BMI differences among different groups.

## Materials and Methods

### Participants and Procedure

Only volunteer participants who were 18 years or older were recruited and completed an internet questionnaire via the professional survey website, Wenjuan xing. Respondents were told that the questionnaire was about sex and eating, that it was entirely anonymous, and that they would receive feedback regarding their survey results. This study was approved by the Ethics Committee of Southwest University of China. All participants gave online informed consent before filling in the questionnaire.

The sample comprised two-hundred and sixty-one Chinese people (136 males and 125 females) from 88 provinces/regions of China. The mean age was 30.46 years (*SD* = 6.788) and ranged from 18 to 59 years old. Two-hundred and twenty-eight (87.4%) respondents were employed, 29 (11.1%) were students, and four (1.5%) were unemployed. Seven (2.7%) participants reported a junior high school education or less, 19 (7.3%) a senior high school education, 196 (75.1%) a college education, and 39 (14.9%) postgraduate education or higher. Twenty-two (8.4%) had never been in a relationship, 25 (9.6%) were currently single, 50 (19.2%) were unmarried but in a relationship, and 164 (62.8%) were married. Two-hundred and fifty-four (97.3%) participants reported they were heterosexual, six (2.3%) bisexual, and one (0.4%) respondent reported “other”; no one reported that they were homosexual. Regarding ethnicity, 256 (98.1%) were Han and 5 (1.9%) an ethnic minority. No religion was reported by 217 (83.1%) individuals, 33 (12.6%) reported Buddhism, eight (3.1%) Christianity, one (0.4%) Islam, and two (0.8%) reported other religions.

### Measures

#### Wanting for Sex

Lippa (2006, 2007) developed a sex drive scale, which assesses respondents’ desire for sex, their frequency of sexual activity, the extent to which they think and fantasize about sex, and their evaluation of how rewarding sex is for them. The sex drive scale (Lippa, 2006) includes five items: (1) I have a strong sex drive, (2) I frequently think about sex, (3) it doesn’t take much to get me sexually excited, (4) I think about sex almost every day, (5) sexual pleasure is the most intense pleasure a person can have. Participants rated how much they agreed with these items on a five-point scale (1 = strongly disagree, 5 = strongly agree), and total score was used as the measure of wanting for sex. The internal consistency was good (Cronbach’s α = 0.886; 0.850 for males and 0.900 for females).

In addition, we assessed individuals’ sociosexuality (i.e., individual differences in the willingness to engage in uncommitted sexual activity) using the SOI-R (Penke and Asendorpf, 2008), to analyze the relationship between sociosexual orientation and eating. The SOI-R (Penke and Asendorpf, 2008) consists of nine items, covering three dimensions for which respondents select the option that best describes their own situation using 9-point scales. The dimensions consist of sociosexual behavior, sociosexual attitude, and sociosexual desire. Each item was scored from 1 to 9 and the total score was used as the measure of sociosexual orientation. The internal consistency was 0.700 (0.626 for males and 0.776 for females).

#### Wanting to Eat

Two scales were used to measure general wanting to eat. First, the RED scale was used to measure basic, non-pathological eating drive (Epel et al., 2014). This scale includes nine items. Participants were asked to rate to what extent they agreed with each statement using a 5-point Likert scale ranging from 1 (strongly disagree) to 5 (strongly disagree). The total score of this scale was used a measure of wanting to eat. The internal consistency was 0.916 (0.912 for males and 0.915 for females).

In addition, we used the 21-item FCQ-T to measure general, but not specific, food cravings (Nijs et al., 2007). Participants were asked to rate their agreement with each item on a scale from 1 (strongly disagree) to 6 (strongly agree). The total score of this questionnaire was also used as a measure of wanting to eat. Cronbach’s alpha was 0.969 (0.968 for males and 0.965 for females).

#### Liking for Sex and Eating

Both liking for sex and eating were measured via one item each: participants rated to what extent they agreed with the statement “how much pleasantness can you can feel from eating/sex” using a 7-point Likert scale (1 = Very little, 7 = A great deal).

#### Emotional Sex Cravings (and Sexual Activity) and Emotional Food Cravings (and Eating Behavior)

One dimension of the FCQ-T (Nijs et al., 2007) is emotional food cravings, consisting of four items (e.g., “I crave foods when I’m upset”). Participants were asked to rate how much they agreed with each statement using a 6-point Likert scale ranging from 1 (strongly disagree) to 6 (strongly disagree). The total score was used as an emotional food craving measure.

The emotional eating scale of the Three-Factor Eating Questionnaire-R18 (Karlsson et al., 2000) was used to assess emotional eating behavior. This scale consists of three items (e.g., “when I feel anxious, I find myself eating”). These items were rated on a four-point scale ranging from 1 (does not describe me at all) to 4 (describes me exactly). The total score was used as an emotional eating behavior variable.

The above two scales were modified to measure emotional sex cravings and emotional sexual activity. The food items were replaced with sex-related items, while response and scoring methods remained the same. The total scores of the two questionnaires were used as an emotional sex craving measure and emotional sexual activity measure, respectively. The internal consistency for emotional food cravings was 0.935 (0.940 for males and 0.921 for females), 0.844 for emotional eating behavior (0.867 for males and 0.799 for females), 0.916 for emotional sex cravings (0.891 for males and 0.920 for females), and 0.863 for emotional sexual activity (0.839 for males and 0.858 for females).

### Data Analyses

Initially, data were analyzed using SPSS version 16.0. We checked the data for normality and for outliers before analysis. We examined six correlations between sex and eating. After Bonferroni correction to control the risk of type I error, the threshold *p*-value for significance was adjusted to 0.00833 (i.e., 0.05 divided by 6). Additionally, correlation coefficients were compared between sexes. All correlation coefficients were transformed into Fisher’s *Z*-values before comparison using SPSS syntax. Second, to explore models of wanting (liking) for sex and eating, LPA was used. LPA allows exploration of latent constructs underlying psychology and behavior, with advantages over other, traditional, techniques such as cluster analysis (Pastor et al., 2007; Armour et al., 2011). In this research, we used Mplus version 6.12 to perform LPA for males and females separately. To explore wanting model, indicator variables for LPA analysis included wanting for sex (total scores of sex drive scale) and wanting to eat (total scores of FCQ-T). We investigated the plausibility of 2-, 3-, 4-, 5-class solutions and determined the best model fit. Model fit was evaluated using the LMRT, the AIC, aBIC, and the entropy. The LMRT compares the fit of one target model to a model having one fewer class (such as a 2-class model vs. a 1-class model). If *p*-values are less than 0.05, this indicates that the higher class solution fits better. Both lower AIC and aBIC indicate a better model. Finally, the closer the entropy value is to 1, the better the model is. To explore liking model, a similar LPA analysis was conducted for the two sexes. Indicator variables were liking for sex (total scores of the liking sex item) and liking for eating (total scores of the liking to eat item). We investigated the plausibility of 2-, 3-, 4-, and 5-class solutions and determined the optimal model fit. Subsequently, MANOVA was used to explore the differences in demographic characteristics among different wanting (liking) groups.

## Results

### Correlations Between Wanting Sex and Wanting to Eat

We tested the correlations between the total scores of the sex drive scale and RED scale and between the total scores of the sex drive scale and FCQ-T to explore the relationship between wanting sex and wanting to eat. There were significant positive correlations only for males. Total scores of the sex drive scale and the RED scale were not significantly correlated overall (*r* = 0.151, *p* = 0.014), and were correlated for males but not females (males: *r* = 0.247, *p* = 0.004; females: *r* = 0.213, *p* = 0.017; sex drive, males: *M* = 18.89, *SD* = 3.91; females: *M* = 16.02, *SD* = 4.57; RED, males: *M* = 25.72, *SD* = 8.33; females: *M* = 28.88, *SD* = 8.00). Total scores of the sex drive scale and FCQ-T were also not significantly correlated overall (*r* = 0.126, *p* = 0.043), with a significant correlation for males (*r* = 0.269, *p* = 0.002; food craving: *M* = 70.71, *SD* = 25.32) but not females (*r* = 0.171, *p* = 0.056; food craving: *M* = 82.42, *SD* = 23.00; **Table 1**).

**Table 1:** Correlations between sex and eating variables.

Sex and eating variables	Total	Male	Female
Wanting for sex and eating			
*Sex drive and RED scale*	0.151	0.247*	0.213
*Sex drive and FCQ-T*	0.126	0.269*	0.171
Liking for sex and eating	0.250*	0.365*	0.200
Sociosexuality orientation and wanting to eat (Spearman’s correlation coefficients)			
*SOI-R and RED scale*	0.168*	0.197	0.308^∗^
*SOI-R and FCQ-T*	0.089	0.164	0.258^∗^
Sociosexuality orientation and liking for eating	0.002	0.177	0.041
Emotional cravings for sex and food	0.257*	0.480*	0.243^∗^
Emotional sexual activity and emotional eating behavior	0.269*	0.439*	0.255^∗^

*^∗^*P* < 0.0083*.

### Correlations Between Liking Sex and Liking Eating

Scores for liking for sex were significant positively related to scores for liking eating overall (*r* = 0.250, *p* < 0.001), and this correlation was significant only in males (males: *r* = 0.365, *p* < 0.001; females: *r* = 0.200, *p* = 0.026; liking for sex, males: *M* = 5.46, *SD* = 1.37; females: *M* = 5.21, *SD* = 1.54; liking for eating, males: *M* = 4.48, *SD* = 1.66; females: *M* = 5.32, *SD* = 1.54; **Table 1**).

### Correlations Between Sociosexual Orientation and Wanting to Eat

The correlation between sociosexual orientation and wanting to eat varied according to the different scales. There was a significant positive correlation between total scores of SOI-R and RED scale (ρ = 0.168, *p* = 0.006), while total scores of SOI-R were not significantly correlated with total scores of FCQ-T (ρ = 0.089, *p* = 0.151). Correlations between total scores of SOI-R and RED scale or FCQ-T were significant only in females (SOI-R, males: ρ = 0.197, *p* = 0.021; females: ρ = 0.308, *p* < 0.001; RED, males: ρ = 0.164, *p* = 0.057; females: ρ = 0.258, *p* = 0.004).

### Correlations Between Sociosexual Orientation and Liking for Eating

There were no significant correlations between total scores of SOI-R and liking eating (overall: ρ = 0.002, *p* = 0.971; males: ρ = 0.177, *p* = 0.039; females: ρ = 0.041, *p* = 0.651).

### Correlations Between Emotional Sex Cravings (or Sexual Activity) and Emotional Food Cravings (or Eating Behavior)

Overall, there was a significant positive correlation between emotional sex craving scores and emotional food craving scores (*r* = 0.257, *p* < 0.001), and the two sexes exhibited significant correlations (males: *r* = 0.480, *p* < 0.001; females: *r* = 0.243, *p* = 0.006; emotional sex craving, males: *M* = 15.32, *SD* = 5.50; females: *M* = 11.31, *SD* = 5.75; emotional food craving, males: *M* = 12.84, *SD* = 5.93; females: *M* = 15.62, *SD* = 5.33). This correlation was stronger for males than for females (*p* = 0.03). There was also an overall significant positive correlation between emotional sexual activity and emotional eating behavior (*r* = 0.269, *p* < 0.001), with significant correlations for both males and females (males: *r* = 0.439, *p* < 0.001; females: *r* = 0.255, *p* = 0.004; emotional sexual activity, males: *M* = 8.06, *SD* = 2.67; females: *M* = 6.26, *SD* = 2.77; emotional eating behavior, males: *M* = 6.99, *SD* = 2.74; females: *M* = 8.02, *SD* = 2.42). The correlation coefficient for males was greater than for females and this difference approached significance (*p* = 0.09; **Table 1**).

### Wanting for Sex and Eating Model

For females, the LMRT was not significant for the first time for the 4-class solution (LMRT = 11.903, *p* > 0.05), which indicates that the 4-class solution was not better than the 3-class solution. The 3-class solution was better than the 2-class solution according to the LMRT value (LMRT = 14.858, *p* < 0.05). Although the 3-class solution had smaller entropy than the 2-class solution, both AIC and aBIC were smaller than the 2-class solution. Taken together, these results suggest that the 3-class solution was optimal (**Table 2**).

**Table 2:** Model fit indexes for the 2-, 3-, 4-, and 5-class solution for wanting in females.

	AIC	aBIC	Entropy	LMRT
2-class	1864.40	1862.06	0.807	19.25, *p* < 0.01
3-class	1854.51	1851.18	0.744	14.858, *p* < 0.05
4-class	1847.79	1843.45	0.787	11.903, *p* > 0.05
5-class	1847.71	1842.36	0.735	32.673, *p* > 0.05

Class 1 (45.6%) was typified by individuals with the lowest level of wanting sex and moderate levels of want to eat. This subgroup we named the *low wanting for sex* group. Class 2 (28.8%) consisted predominantly of individuals who had the highest level of wanting for both sex and eating. Therefore, this group was named the *high wanting* group. Class 3 (25.6%) primarily included low levels of wanting to eat and moderate levels of sexual desire. This group was named the *low wanting for eating* group (**Table 3**).

**Table 3:** Wanting for sex and eating by class (mean, SD).

	Female			Male	
Wanting Scale	Class 1 *n* = 57	Class 2 *n* = 36	Class 3 *n* = 32	Class 1 *n* = 72	Class 2 *n* = 64
Sex drive scale	14.2 (3.8)	18.9 (3.6)	16.0 (5.1)	17.7 (4.2)	20.2 (3.1)
FCQ-T	85.0 (8.5)	107.3 (6.8)	49.9 (10.6)	50.4 (13.2)	93.6 (13.1)
RED scale	28.6 (5.3)	37.4 (2.9)	19.7 (4.9)	20.2 (5.3)	32.0 (6.5)

For males, the LMRT was not significant for the 3-class solution (LMRT = 8.430, *p* > 0.05), indicating that the 3-class solution was no better than the 2-class solution. Additionally, entropy began to decrease for the 3-class solution. Taken together, these results led us to consider the 2-class solution the best model fit (**Table 4**).

**Table 4:** Model fit indexes for the 2-, 3-, 4-, and 5-class solution for wanting in males.

	AIC	aBIC	Entropy	LMRT
2-class	2007.977	2006.221	0.732	23.961, *p* < 0.001
3-class	2004.975	2002.467	0.668	8.430, *p* > 0.05
4-class	2007.681	2004.421	0.677	3.084, *p* > 0.05
5-class	2006.152	2002.139	0.73	7.051, *p* > 0.05

Class 1 (52.9%) was typified by individuals having lower level of wanting for sex and eating. This subgroup was named the *low wanting* group. Class 2 (47.1%) consisted predominantly of individuals with higher levels of wanting for both sex and eating, leading us to assign name this class the *high wanting* group (**Table 3**).

In addition, MANOVA was conducted to test whether there were differences in demographic characteristics among these groups. We tested the effect of class on age, height, weight, and BMI. For females, there were no significant differences in demographic characteristics among the three groups (all *p*s > 0.05). For males, there was a significant difference in age between the two groups [*F*(1,134) = 4.341, *p* = 0.039, η^2^ = 0.031]: the low wanting group (*M* = 29.5, *SD* = 6.72) was younger than the high wanting group (*M* = 32.1, *SD* = 8.04). Additionally, the low wanting group (*M* = 172.3, *SD* = 5.64) was shorter than the high wanting group (*M* = 173.9, *SD* = 5.26), a difference which approached significance [*F*(1,134) = 2.859, *p* = 0.093, η^2^ = 0.021].

### Liking for Sex and Eating Model

For females, non-significant LMRT occurred for the first time for the 3-class solution, indicating that the 3-class solution was no better than the 2-class solution. Further, the 2-class solution had the greatest entropy. As such, the 2-class solution was considered optimal (**Table 5**).

**Table 5:** Model fit indexes for the 2-, 3-, 4-, and 5-class solution for liking in females.

	AIC	aBIC	Entropy	LMRT
2-class	883.379	881.042	0.964	50.342, *p* < 0.001
3-class	877.775	874.436	0.891	10.855, *p* > 0.05
4-class	867.438	863.098	0.910	15.282, *p* < 0.05
5-class	866.524	861.183	0.845	6.467, *p* > 0.05

Class 1 had a greater level of liking for sex and eating than class 2, so the former was named the *high liking* group (88%) and the latter the *low liking* group (12%; **Table 7**).

For males, LMRT was not significant for the 4-class solution (LMRT = 3.734, *p* > 0.05), signifying that this model was no better than the 3-class solution. LMRT was significant for the 3-class solution (LMRT = 18.548, *p* < 0.01), denoting that the 3-class solution was better than the 2-class solution. In addition, the 3-class model had the smallest AIC, relatively small aBIC, and the largest entropy. Overall, these results indicated the 3-class solution was the best model (**Table 6**).

**Table 6:** Model fit indexes for the 2-, 3-, 4-, and 5-class solution for liking in males.

	AIC	aBIC	Entropy	LMRT
2-class	957.538	955.783	0.871	46.180, *p* < 0.01
3-class	929.716	927.208	0.892	31.673, *p* < 0.001
4-class	929.846	926.586	0.738	5.497, *p* > 0.05
5-class	927.939	923.926	0.826	7.405, *p* > 0.05

Class 1 (16.9%) had a low level of liking for sex and eating and this group was named the *low liking* group. Class 2 (10.3%) consisted predominantly of individuals who had the highest level of liking for sex and the lowest level of liking for eating. Therefore, this group was named the *high liking for sex* group. Class 3 (72.8%) primarily included the highest level of liking for eating and moderate levels of liking for sex. As such, this named the *high liking for eating* group (**Table 7**).

**Table 7:** Liking for sex and eating by class (mean, SD).

	Female		Male		
Liking Scale	Class 1 *n* = 110	Class 2 *n* = 15	Class 1 *n* = 23	Class 2 *n* = 14	Class 3 *n* = 99
Liking for sex	5.3 (1.5)	4.3 (1.9)	3.3 (1.1)	6.6 (0.6)	5.8 (1.0)
Liking for eating	5.8 (0.9)	1.9 (0.8)	2.3 (0.8)	2.0 (0.9)	5.3 (0.9)

A MANOVA was again conducted to test whether there were demographic characteristic differences among these groups. For females, there were no significant differences in these demographic characteristics (all *p*s > 0.05). For males, there was a significant difference in height among these three groups [*F*(2,133) = 3.348, *p* = 0.038, η^2^ = 0.048]. The low liking group (*M* = 170.5, *SD* = 5.18) was shorter than the high liking for sex group (*M* = 173.7, *SD* = 5.29).

## Discussion

We studied the general association between sex and eating from a reward perspective. We found that (1) there were significant positive correlations between wanting (and liking) for sex and eating only in males; (2) sociosexual orientation was positively correlated with females’ wanting to eat; (3) emotional sex cravings (or sexual activity) were significantly related to emotional food cravings (or eating behavior), and the relationship was stronger in males than in females; (4) each sex had a different wanting (liking) model.

That significant correlations between wanting (and liking) for sex and eating were present only in males indicates that sex and eating might be more consistently comparable in males than in females. That is, it is possible that sex and eating play a more equal role in males than in females. Sex and eating are related to reproduction and personal survival; can we thus conclude that these two primary needs are more equivalently important to males than females? Currently, we cannot answer this question; future research is needed. However, of relevance is the work of Herzog et al. (1984), who found that, compared with their female counterparts, males with anorexia and bulimia are more like to report sexual isolation, sexual inactivity, and conflicted homosexuality. We suggest the basis of this phenomenon may be that sex and eating have more balanced importance in males than in females. Therefore, if one of these (sex or eating) is dysfunctional, the other (eating or sex) is more likely to also display abnormal or non-traditional characteristics in males than in females. Finally, many studies have explored sex and eating separately. These studies indicate that sex and eating share some brain areas (for a review, see Georgiadis and Kringelbach, 2012). Unfortunately, few brain imaging studies have explored sex and eating simultaneously, and further, gender differences in this context have been seldom investigated. However, based on existing studies and our results, it would be of interest to see whether gender differences are evident in these related brain areas. For example, would it be possible that less variation in brain activity for sex and food stimuli for males than females?

There was a significant positive correlation between sociosexual orientation and wanting to eat only in females. This indicates that more sexually unrestricted female individuals may have a stronger desire to eat. Existing studies have found that females with bulimia nervosa tend to exhibit promiscuous sexual behaviors, have more sexual activity, and engage in more sexual intercourse than controls (Wiederman et al., 1996; Kaltiala-Heino et al., 2003; Culbert and Klump, 2005). Although our study focused on a community sample, and not people with eating disorders, our results were consistent with these existing results to some extent. Further, we found no significant relationships between sociosexual orientation and liking to eat for both males and females. These two results indicate that sociosexual orientation may be related only to females’ wanting to eat.

Eating disorders are frequently accompanied by abnormal sexuality, non-traditional sex roles, and non-heterosexual orientation. Accordingly, we explored the relationship between eating and sex under emotional stress. We found a positive correlation between general emotional sex cravings (or sexual activity) and general emotional food cravings (or eating behavior). To manage negative mood or release stress, sex and eating may play the same role for some individuals. In addition, the correlation with emotional cravings was of higher magnitude in males than in females, while the correlation with emotional behavior in males was higher than in females, albeit falling just outside significance. This indicates that males’ emotional cravings for sex and food, but not emotional behaviors, were more comparable and consistent than in females. However, because we did not assess individuals’ actual behaviors, this almost significant result remains to be tested further via additional experiments that directly measure behavior. As mentioned above, males with anorexia and bulimia are more likely than their female counterparts to report sexual isolation, sexual inactivity, and conflicted homosexuality (Herzog et al., 1984). In our study’s community sample we found that sex and eating may play a more equal role in managing negative emotions or stress in males than females, which is broadly consistent with the research on sexual behavior in individuals with eating disorders, as noted earlier. Therefore, males being treated for eating disorders (or abnormal sexuality) should also be screened regarding their sexual function (or eating behavior).

The wanting model in females had three types: high wanting, low wanting for eating, and low wanting for sex. The wanting model in males had two types: high wanting and low wanting. For males, the statement “people who like eating may have a high sex drive” may be reasonable, and males who have a limited desire to eat may also have little desire for sex. However, this is not the case for females. Females with moderate levels of wanting to eat may have the lowest levels of sexual desire. There were two liking models in females: high liking and low liking; whereas there were three liking models in males: high liking for sex, high liking for eating, and low liking. A woman who receives little pleasure from eating may also receive little pleasure from sex. In contrast, a man who receives little pleasure from eating may receive little pleasure from sex, or considerable pleasure from sex. The pleasure received from sex will not be low for individuals who receive great pleasure from eating: females receive considerable sexual pleasure, and males, moderate pleasure. Both sex and eating are important in life; eating is more vital than sex for individuals (individual survival) while sex is more necessary than eating for the species (reproduction and continuation of the species). Although both sex and eating are important, our results indicate that one model cannot describe all individuals. For example, some people have high level of desire to eat but low desire for sex, some have a high level of desire for sex and eating, while others may want sex and food relatively little. Is the individual survival motivation more important than the reproduction motivation for some people, while the opposite is true for others? Is this difference displayed in individuals’ mating motives or behaviors related to individual survival? We found that the high wanting group was older and taller than the low wanting group in males, and the high liking for sex group was taller than the low liking group in males. It seems that females prefer old and tall males rather than young and short males (Brooks and Kemp, 2001; Salska et al., 2008). Therefore, we were curious about whether there was any difference in the chance of mating among different groups in males. Future research could explore further such differences related to sexuality, mating, and individual survival among different groups. In addition, non-drug reward (such as shopping, eating, sexual behavior, and gambling) can alter neural plasticity, similarly to drug-based reward (Winder et al., 2002; Kauer and Malenka, 2007; Lüscher and Bellone, 2008; Thomas et al., 2008; Olsen, 2011). Therefore, individuals with different levels of wanting for sex and eating may have differences in their underlying neural structures, following the influence of different reward schedules over a long period of time. Therefore, further studies might explore brain structure or personality differences among different wanting (liking) groups. Finally, we explored models in a community sample, leaving the question open as to what the models would be for eating disorder groups or sexual dysfunction groups.

The primary shortcoming of our research was that all correlation coefficients were relatively modest. The measures used might underlie this result: questionnaires and online surveys have limitations that could be addressed via future laboratory-based research. However, given that our objective was to provide a novel, preliminary analysis of the general relationship between sex and eating, that we found any relationships is consequential. Second, Dewitte (2015) explored gender differences in wanting and liking sex by explicit and implicit measurements. Thus, we do not know if the relationship between sex and eating would display the same characteristics if assessed using implicit measures. Third, considering impulsivity is related to binge eating and risky sexual behavior, it is possible that impulsivity may be of relevance to this field. We did not control this variable, and future research could usefully explore the role of impulsivity. Fourth, we do not know whether there were differences in correlations between sex and eating measures among sexually active and inactive individuals, or between sexually experienced and inexperienced individuals. Finally, our sample consisted of Chinese people. Considering the unique Chinese cultural context pertaining to food and sex, it may be that the relationship between sex and eating varies with different cultural backgrounds. Future studies should consider these issues.

The correlation between sex and eating has received some recent attention, including a realization of the many similarities between sex and eating. For instance, sex and eating are both consummatory behaviors that have rhythmicity; for both, novelty is motivating; and, learning and prediction are important in both behaviors (Georgiadis and Kringelbach, 2012). Given the associations between sex and eating in terms of behavior, psychology, and underlying brain representations, future research could explore whether these correlations infiltrate social and cultural contexts through evolution. Future research might explore social and cultural correlates of sex and eating from a metaphorical perspective. For example, one might ask whether certain food stimuli engender sexual thoughts while others do not, and whether any such phenomena differ across cultures.

## Author Contributions

YK carried out the experimental work and the data collection, interpretation and drafted the manuscript. LZ and YZ participated in the design and coordination of experimental work and interpretation, and revised the manuscript. All authors read and approved the final manuscript.

## Conflict of Interest Statement

The authors declare that the research was conducted in the absence of any commercial or financial relationships that could be construed as a potential conflict of interest.

## ABBREVIATIONS

AIC: akaike information criterion
aBIC: sample size-adjusted Bayesian information criterion
FCQ-T: Trait General Food Cravings Questionnaires
LMRT: Lo-Mendell-Rubin adjusted likelihood ratio test
LPA: latent profile analysis
RED: Reward-Based Eating Drive
SOI-R: Sociosexuality Orientation Inventory-Revised.
